# Structure of a Hydrated Sulfonatotitanyl(IV) Complex in Aqueous Solution and the Dimethylsulfoxide Solvated Titanyl(IV) Ion in Solution and Solid State

**DOI:** 10.1007/s10953-017-0581-3

**Published:** 2017-01-27

**Authors:** Daniel Lundberg, Ingmar Persson

**Affiliations:** Department of Molecular Sciences, Swedish University of Agricultural Sciences, P.O. Box 7015, SE-750 07 Uppsala, Sweden

**Keywords:** Titanyl(IV), Aqueous solution, Sulfate, Structure, Dimethylsulfoxide (DMSO)

## Abstract

The coordination chemistry of oxotitanium(IV) or titanyl(IV), TiO^2+^, has been studied in solution by X-ray methods. The titanyl(IV) ion hydrolyzes easily in aqueous systems to solid titanium dioxide as long as it is not stabilized through complexation. In this study the structures of the hydrated bissulfatotitanyl(IV) complex and the dimethylsulfoxide (DMSO) solvated titanyl(IV) ions have been determined. In isolated monomeric titanyl complexes titanium(IV) binds strongly to a doubly bound oxo group at ca. 1.64 Å, to four ligands in the equatorial plane almost perpendicular to the Ti=O bond at ca. 2.02 Å, and there is one weakly bound ligand, *trans* to the Ti=O bond, at ca. 2.22 Å, for oxygen donor ligands; the O=Ti–O_eq_ bond angles are 95°–100°. The structure of the DMSO solvated titanyl(IV) ion in the solid state is maintained in DMSO solution.

**Electronic supplementary material:**

The online version of this article (doi:10.1007/s10953-017-0581-3) contains supplementary material, which is available to authorized users.

## Introduction

The chemistry of titanium(IV) is strongly dominated by different forms of titanium dioxide, natural rutile, anatase and brookite, two high-pressure forms, akaogiite and TiO_2_ II, and solid titanate compounds. In these compounds titanium(IV) binds octahedrally six oxygens at a mean Ti–O bond distance of ca. 1.96 Å [1]. Titanium dioxide, in its different forms, is produced in millions of tons annually to be used in wide ranges of technical applications as, e.g., pigments and nano-sized materials [2]. Titanium dioxide-based titanates are formed at high temperatures. Orthotitanates and metatitanates have the formulas M_2_TiO_4_ and MTiO_3_, respectively, where M is a divalent metal ion. Their names are somewhat misleading as they almost never contain ${\text{TiO}}_{4}^{4 - }$ or ${\text{TiO}}_{3}^{2 - }$ units, respectively. One exception is Ba_2_TiO_4_ with an identifiable ${\text{TiO}}_{4}^{4 - }$ unit with a mean Ti–O bond distance of 1.81 Å [3–5]. Also, the metatitanate Rb_2_TiO_3_ is four-coordinate in a chain-structure with a mean Ti–O bond distance of 1.81 Å [6, 7]. The orthotitanates, M_2_TiO_4_, have the spinel structure, while the metatitanates have either ilmenite (FeTiO_3_) or perovskite (CaTiO_3_) structures [1, 8]. The ortho- and metatitanates have network structures with titanium being octahedrally surrounded by six oxygen atoms at ca. 1.96 Å [1].

Titanium dioxide can only be dissolved in concentrated acids where the anion is able to form reasonably stable complexes with the oxotitanium(IV) ion, or, more commonly, the titanyl(IV) ion, TiO^2+^, such as in sulfuric acid solution. The titanyl(IV) ion is only stable and soluble in aqueous solution as complexes with, e.g., sulfate, phosphate, oxalate, citrate, lactate, malate or tartrate ions [9]. The number of reported crystal structures with an isolated titanyl(IV) complex is very limited, including Ba_2_[TiO(Si_2_O_7_)_2_] [10], Sr_2_[TiO(PO_4_)_2_] [11] (C(NH_2_)_3_)_4_[TiO(CO_3_)_3_]·2H_2_O [12] and [TiO(OS(CH_3_)_2_)_5_]Cl_2_ [13, 14]. In Ba_2_[TiO(Si_2_O_7_)_2_] and [TiO(OS(CH_3_)_2_)_5_]Cl_2_ titanium(IV) binds strongly to an oxogroup at ca. 1.64 Å, to four oxygens atoms in the equatorial plane perpendicular to the Ti=O bond at ca. 2.03 Å, and in the *trans* position to the Ti=O bond an oxygen donor ligand is weakly coordinated at ca. 2.22 Å, or the position may be empty as in Ba_2_[TiO(Si_2_O_7_)_2_]. In Sr_2_[TiO(PO_4_)_2_] the Ti=O bond distance is longer, 1.747 Å, and the ligand in the *trans* position to the Ti=O bond is equal in length to the equatorials bonds [11]. Titanium(IV) is seven-coordinate in (C(NH_2_)_3_)_4_[TiO(CO_3_)_3_]·2H_2_O with one of the oxygens in each bidentately bound carbonate ion at significantly shorter Ti–O bond distance than the other, mean values at 2.08 and 2.18 Å, respectively. A structure with a hydrolyzed isolated hexameric dimethylsulfoxide (DMSO) solvated titanyl(IV) structure has been reported as well, [Ti_6_O_4_(OS(CH_3_)_2_)_12_]Cl_4_·5((CH_3_)_2_SO)·0.5H_2_O [13].

The aim of this study was to determine the structure of the hydrated and DMSO solvated titanyl(IV) in aqueous and DMSO solutions, respectively, as no structural studies of solvated titanyl(IV) ions or complexes in solution have been reported. The titanyl(IV) ion displays a range of different bond distances to neutral ligands besides the short Ti=O double bond, vide supra. In spite of several attempts it has not been possible to stabilize the hydrated titanyl(IV) ion at concentrations allowing structure determination by X-ray methods to be applied. The titanyl(IV) ion hydrolyzes easily in aqueous systems to solid titanium dioxide unless it is not stabilized through complexation. However, complexation with, e.g., sulfate ions stabilizes titanyl(IV) substantially allowing the structure of a hydrated [TiO(SO_4_)_*n*_]^(2*n*−2)−^ complex to be studied in dilute sulfuric acid solution. Furthermore, in this work the structure of the DMSO solvated titanyl(IV) ion has been determined crystallographically as the trifluoromethanesulfonate salt and in DMSO solution to allow comparisons between a solvate and a complex in aqueous solution.

## Experimental Section

### Chemicals Used

Dimethylsulfoxide, (CH_3_)_2_SO (Merck, puriss), DMSO, was freshly distilled under vacuum and over calcium hydride, CaH_2_ (Fluka, puriss), before use. Titanyl(IV) sulfate, TiOSO_4_·H_2_O (Aldrich), barium carbonate, BaCO_3_ (Fluka, p.a.), and trifluoromethanesulfonic acid, CF_3_SO_3_H (Fluka, p.a.), were used as purchased.

### Preparation of Salts and Solutions

Barium trifluoromethanesulfonate, Ba(CF_3_SO_3_)_2_, was prepared by dropwise addition of trifluoromethanesulfonic acid to a slurry of barium carbonate under stirring until a clear solution was obtained. The obtained solution was cooled to room temperature and filtered, and thereafter put into an oven at 450 K to boil off water and excess acid. The obtained white powder was ground into a fine powder and stored in the oven at 450 K.

Pentakis(dmso)titanyl(IV) trifluoromethanesulfonate, [TiO(OS(CH_3_)_2_)_5_](CF_3_SO_3_)_2_, **1**, was prepared by mixing equimolar DMSO solutions of barium trifluoromethanesulfonate and titanyl(IV) sulfate, the mixture was stirred for 10 min, and the barium sulfate formed was filtered off. The volume was reduced until compound **1** started to precipitate, and the mixture was then refrigerated for further crystallization.

The aqueous solution for the LAXS experiment was a commercial titanyl(IV) sulfate solution (Sigma–Aldrich) and used as purchased after dilution with deionized water. The DMSO solution of titanyl(IV) trifluoromethanesulfonate was prepared by dissolving **1** in freshly distilled DMSO. The compositions of the studied solutions are summarized in Table 1.

**Table 1:** Compositions (in mol·dm^−3^), densities (*ρ*), and linear absorption coefficients (*μ*) of the aqueous solution of titanyl(IV) (hydrogen)sulfate acidified with sulfuric acid, and the dimethylsulfoxide (DMSO) solution of titanyl(IV) trifluoromethanesulfonate studied by LAXS and EXAFS, respectively

Sample	[TiO^2+^]	[X^−^]	[solvent]	*ρ*/g·cm^−3^	*μ*/cm^−1^
${\text{TiO(SO}}_{4} )_{2}^{2 - }$ in water^a,b^	1.454	8.594	27.388	1.400	5.64
TiO(CF_3_SO_3_)_2_ in DMSO^b^	1.300	2.600	11.272	1.423	9.93

^a^LAXS

^b^EXAFS

### Single Crystal X-ray Diffraction

Data were collected on a Bruker SMART CCD 1 K diffractometer at ambient temperature, Table 2. The crystal was mounted in a glass capillary, which was sealed by flame immediately after mounting. The structure was solved by standard direct methods in the SHELXL 2014/7 program package [15] and refined isotropically by full matrix least-squares on *F* ^2^, and finally in the anisotropic approximation on all non-hydrogen atoms. Hydrogen atoms were refined using a riding model. All structure solutions were performed with the SHELXL 2014/7 programs in PC version [15]. One of the coordinating DMSO ligands was refined with its sulfur atom in two positions, denoted as S6 and S6b, with partial occupancies of 88.6 and 11.4%, respectively. Also, one of the two trifluoromethanesulfonate counter ions was refined with two sets of fluorine and oxygens atoms with partial occupancies of 54 and 46%, respectively. Selected crystal and experimental data are summarized in Table 2. The atomic coordinates, bond distances and angles are available in a Crystallographic Information File (CIF) in the Supplementary Material section, Table S1. The structure has been deposited to the Cambridge Structure database with CCDC code 1505833.

**Table 2:** Crystallographic data and structure refinement details for pentakis(dimethylsulfoxide)-titanyl(IV) trifluoromethanesulfonate, **1**

1	
Formula	[TiO(CH_3_)_2_SO)_5_](CF_3_SO_3_)_2_
*M*_W_/g·mol^−1^	752.68
Diffractometer system	Bruker Smart CCD
Radiation (*λ*/Å)	0.71073
Crystal system	Triclinic
Space group	P-1 (No. 2)
*a*/Å	10.198(5)
*b*/Å	13.495(6)
*c*/Å	13.797(6)
*α*/°	99.002(9)
*β*/°	110.814(8)
*γ*/°	110.231(8)
*V*/Å^3^	1578.5(13)
*T*/K	295(2)
*Z*	2
*D*_c_/g·cm^−1^	1.584
*F* ^2^	772
*µ*/mm^−1^	0.816
Crystal size/mm	0.60 × 0.20 × 0.15
*θ* range/°	2.93–24.93
Index ranges	–11 ≤ *h* ≤ 11
	–14 ≤ *k* ≤ 14
	–7 ≤ *l* ≤ 15
Measured reflections	5575
Unique reflections	3327
Data/restraints/params	4170/24/410
Goodness of fit	1.092
Refinement method	Full-matrix least-sq. *F* ^2^
Final *R*_1_, w*R*_2_ [*I* > 2σ(*I*)]^a^	0.0721, 0.2031
Final *R*_1_, w*R*_2_ [all data]	0.0856, 0.2216
Max. peak/hole eÅ^−3^	0.612/–0.795

^a^ *R* values are defined as: $R_{1} = \sum \left| {\left| {F_{o} } \right|{-}\left| {F_{c} } \right|} \right|/\sum \left| {F_{o} } \right|,wR_{2} = \left[ {{{\sum \left[ {w\left( {F_{o}^{2} {-}F_{c}^{2} } \right)^{2} } \right]} \mathord{\left/ {\vphantom {{\sum \left[ {w\left( {F_{o}^{2} {-}F_{c}^{2} } \right)^{2} } \right]} {\sum \left[ {w\left( {F_{o}^{2} } \right)^{2} } \right]}}} \right. \kern-0pt} {\sum \left[ {w\left( {F_{o}^{2} } \right)^{2} } \right]}}} \right]^{0.5}$

### XAFS: Data Collection

Titanium K-edge X-ray absorption spectra were recorded at the wiggler beam line 4-1 at the Stanford Synchrotron Radiation Lightsource (SSRL). The EXAFS station was equipped with a Si[111] double crystal monochromator. SSRL operated at 3.0 GeV and a current of 97–100 mA in the top up mode. The data collection was performed simultaneously in the transmission mode, using ion chambers with a gentle flow of nitrogen gas, and in the fluorescence mode using a 13-element Ge array solid state detector at ambient temperature. Higher order harmonics were reduced by detuning the second monochromator crystal to 30% of maximum intensity at the end of the scans. The solutions were placed in cells with 1.0 mm Teflon spacers and 6 μm polypropylene foil windows. The energy scale of the X-ray absorption spectra was calibrated by assigning the first inflection point of the K edge of a titanium foil to 4966 eV [16]. For each sample 5 scans were averaged, giving satisfactory data (*k* ^3^-weighted) in the *k*-range 2–13 Å^−1^. The EXAFSPAK program package was used for the data treatment [17].

### EXAFS: Data Analysis

The EXAFS oscillations were extracted from raw averaged data using standard procedures for pre-edge subtraction, spline removal and data normalization. In order to obtain quantitative information of the coordination structure of the metal ions, the experimental *k* ^3^-weighted EXAFS oscillations were analyzed by non-linear least-squares fits of the data to the EXAFS equation, refining the model parameters, number of backscattering atoms, *N*_i_, mean interatomic distances *R*, Debye–Waller factor coefficients, *σ* ^2^, and threshold energy, *E*_o_. Data analysis was performed using the EXAFSPAK program package [17]. Model fitting was performed with theoretical phase and amplitude functions calculated by the ab initio code FEFF (version 7.02) [18]. The standard deviations reported for the obtained refined parameters listed in Tables 3 and 4 are those related to the least-squares refinements and do not include any systematic errors. Variations in the refined parameters obtained using different models and data ranges indicate that the accuracy of the distances given for the separate complexes is within ±0.005–0.02 Å, which is typical for well-defined interactions.

**Table 3:** Mean bond distances, *d*/Å, number of distances, *N*, temperature coefficients, *b*/Å^2^, and the half-height full width, *l*/Å, in the LAXS studies of the hydrated (hydrogen)sulfatotitanyl complex in aqueous solution at ambient room temperature

Species^a^	Interaction	*N*	*d*	*b*	*l*
(Hydrogen)sulfatotitanyl(IV) complex in solution acidified with dilute sulfuric acid					
[Ti(OSO_3_)_2_]^2− a^	Ti=O	1	1.642(5)	0.0019(5)	0.06(1)
	Ti–O_eq_	4	2.025(3)	0.0030(2)	0.077(3)
	Ti–O_ax_	1	2.21(2)	0.021(3)	0.20(2)
	Ti–(O)–S	4	3.513(6)	0.0068(8)	0.117(6)
${\text{SO}}_{4}^{2 - }$(aq)	S–O	4	1.505(4)	0.0044(2)	0.094(2)
	O_SO4_···O_SO4_	4	2.458(7)	0.0072(5)	0.120(4)
O···O^b^	O···O	2	2.834(8)	0.0214(14)	0.207(7)

^a^[Ti(OSO_3_)_2_]^2−^ may also be [Ti(OSO_3_)(OSO_3_H)]^−^ and/or [Ti(OSO_3_H)_2_]

^b^Intermolecular O···O distances including water–water in the aqueous bulk and sulfate oxygen–water

**Table 4:** Number of distances, *N*, mean bond distances, *R*/Å, maximum of bond distance distribution, *R*_m_/Å, Debye–Waller factor coefficients, *σ* ^2^/Å^2^, refined threshold energy, *E*_o_, amplitude reduction factor, *S*_o_^2^, and the error square sum as defined in the EXAFSPAK program package the EXAFS studies of the dimethylsulfoxide (DMSO) solvated titanyl(IV) ion in solution at room temperature

Scattering path	*N*	*d*	*σ* ^2^	*E*_o_	*S*_o_^2^	*F*
(Hydrogen)sulfatotitanyl(IV) complex in sulfuric acid–water						
Ti=O	1	1.646(5)	0.0038(7)	–7.9(1.3)	0.95(9)	11.8
Ti–O_eq_	4	2.036(4)	0.0064(4)			
Ti–O_ax_	1	2.22(2)	0.008(2)			
MS(TiO_4_,sq)	3×4	3.96(4)	0.023(5)			
Ti···S	2	3.493(7)	0.0076(6)			
Ti–O–S	4	3.51(2)	0.009(2)			
DMSO solvated titanyl(IV)ion in DMSO solution						
Ti=O	1	1.646(5)	0.0044(7)	–7.7(1.2)	0.92(9)	17.8
Ti–O_eq_	4	2.035(4)	0.0064(4)			
Ti–O_ax_	1	2.22(2)	0.016(2)			
MS(TiO_4_,eq)	3×4	4.06(4)	0.023(5)			
Ti···S	2	3.193(7)	0.0137(6)			
Ti–O–S	4	3.386(9)	0.0046(9)			
Solid [TiO(dmso)_5_](CF_3_SO_3_)_2_, **1**						
Ti=O	1	1.648(2)	0.0034(7)	–7.6(8)	0.85(9)	10.2
Ti–O	4	2.040(4)	0.0064(4)			
Ti–O_ax_	1	2.23(1)	0.0112(8)			
MS(TiO_4_,eq)	3×4	4.08(4)	0.023(5)			
Ti···S	2	3.200(7)	0.0055(6)			
Ti–O–S	3	3.393(9)	0.0146(9)			

### Large-Angle X-ray Scattering

A large-angle *θ*–*θ* diffractometer was used to measure the scattering of Mo Kα radiation (*λ* = 0.7107 Å) on the free surface of an aqueous solution of titanyl(IV) sulfate acidified with dilute sulfuric acid. The solution was contained in a Teflon cuvette inside a radiation shield with beryllium windows. After monochromatization of scattered radiation, by means of a focusing LiF crystal, the intensity was measured at 450 discrete points in the range 1 < *θ* < 65° (the scattering angle is 2*θ*). A total of 100,000 counts was accumulated at each angle and the whole angular range was scanned twice, corresponding to a statistical uncertainty of about 0.3%. The divergence of the primary X-ray beam was limited by 1° or ^1^/_4_° slits for different *θ* regions with overlapping of some parts of the data for scaling purposes.

All data treatment was carried out by using of the KURVLR program [19] that has been described in detail previously [20]. The experimental intensities were normalized to a stoichiometric unit of volume containing one titanium atom, using the scattering factors f for neutral atoms, including corrections for anomalous dispersion, Δf′ and Δf″ [21], and values for Compton scattering [22]. For a better alignment of the intensity function, a Fourier back-transformation was applied to eliminate spurious (not related to any interatomic distances) peaks below 1.2 Å in the radial distribution function [23]. Least-squares refinements of the model parameters were performed by means of the STEPLR program [24] to minimize the error square sum $U = \, \varSigma w\left( s \right) \times \left[ {i_{\exp } \left( s \right) \, {-}i_{\text {cal}} \left( s \right)} \right]^{2}$.

## Results and Discussion

### Hydrated Sulfonatotitanyl(IV) Complex

**Fig. 1:**
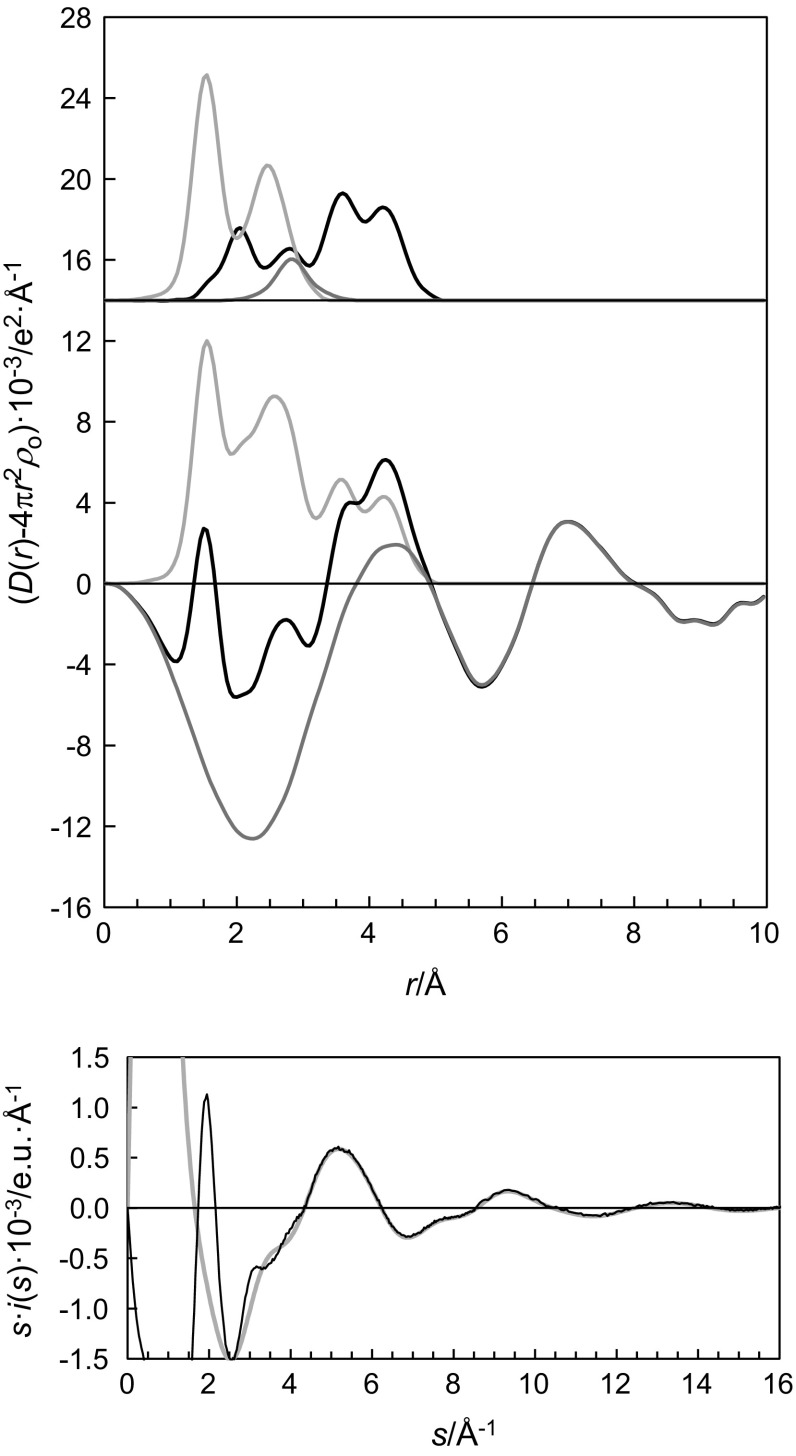
(*Top plot*) LAXS radial distribution *curves* for a 1.454 mol·dm^−3^ acidic aqueous solution of titanyl(IV) (hydrogen)sulfate acidified with dilute sulfuric acid. *Upper part* separate model contributions (offset: 14) of the hydrated *bis*(hydrogen)sulfatotitanyl(IV) complex (*black line*), the hydrated sulfate ion (*grey line*) and aqueous bulk (*dark grey line*). (*Middle plot*) experimental RDF: *D*(*r*) − 4p*r* ^2^ *ρ*_o_ (*grey line*), sum of model contributions (*black line*), and difference (*dark grey line*). (*Bottom plot*) reduced LAXS intensity functions: *s*.*i*(*s*), *black line*; model *s*.*i*_calc_(*s*), *grey line*

The complexation with sulfate ions stabilizes titanyl(IV) substantially, and in this work the structure of the hydrated [TiO(SO_4_)_*n*_]^(2*n*−2)−^ or [TiO(HSO_4_)_*n*_]^(*n*−2)−^ complex has been determined in dilute sulfuric acid; the applied methods cannot distinguish between sulfate and hydrogensulfate ion bound to titanyl(IV). Due to this fact, below we will refer to the ion as (hydrogen)sulfate. Two studies on the complex formation of titanyl(IV) sulfate systems are reported. In one study the formation of two complexes was reported [25, entry 1969BMg], while in the other a very unusual pattern of stability constants with *K*_1_ < *K*_2_ < *K*_3_ is proposed [25, entry 1969VVa]. The experimental radial distribution function (RDF) from the LAXS measurement of the dilute sulfuric acid aqueous solution of titanyl(IV) (hydrogen)sulfate shows peaks at 1.5, 2.75 and 4.3 Å, and shoulders at 2.0 and 3.6 Å, Fig. 1. The strong peak at 1.5 Å corresponds to the S–O bond distance within the (hydrogen)sulfate ion, the broad peak at 2.75 Å corresponds to O···O and O_aq_···O_aq_ distances within the (hydrogen)sulfate ion and the aqueous bulk solution, respectively, and the peak at 4.3 Å to the second hydration sphere, Ti···O_II_. The shoulder at 2.0 Å corresponds to the equatorial Ti–O bond distances, and the shoulder at 3.6 Å to the Ti–(O)–S distance. The structure parameters for the bis(hydrogen)sulfatotitanyl(IV) ion or complex have been refined to 1.642(5) Å for the Ti=O bond, and 2.025(8) Å for the four oxygens in the equatorial plane and to 2.21(5) Å for the oxygen *trans* to the Ti=O bond, Table 3. The Ti–(O)–S distance was refined to 3.51(1) Å showing that the (hydrogen)sulfate ions bind monodentately to titanium with a Ti–O–S bond angle close to linearity. The mean S–O bond distance of the (hydrogen)sulfate ions has been refined to 1.505(2) Å, which is slightly longer than for the hydrated sulfate ion in aqueous solution, 1.495 Å [26]. The reason for this difference could be incomplete hydration as there is not sufficient water in the solution for complete hydration of the (hydrogen)sulfate ions, and those binding to the titanyl(IV) ion have a distorted S–O bond distance distribution. A second hydration sphere to the two water molecules binding to titanium was refined to 4.21(1) Å. This indicates that the composition of the predominant complex in aqueous solution at high (hydrogen)sulfate concentration, according to the LAXS study, is hydrated [TiO(SO_4_)_2_]^2−^, [TiO(SO_4_)(HSO_4_)]^−^, or [TiO(HSO_4_)_2_]. The refined structural parameters from the LAXS measurement are summarized in Table 3, and the experimental and the calculated RDFs are shown in Fig. 1, together with the individual contributions from the respective complexes and ions and intermolecular O···O distances.

**Fig. 2:**
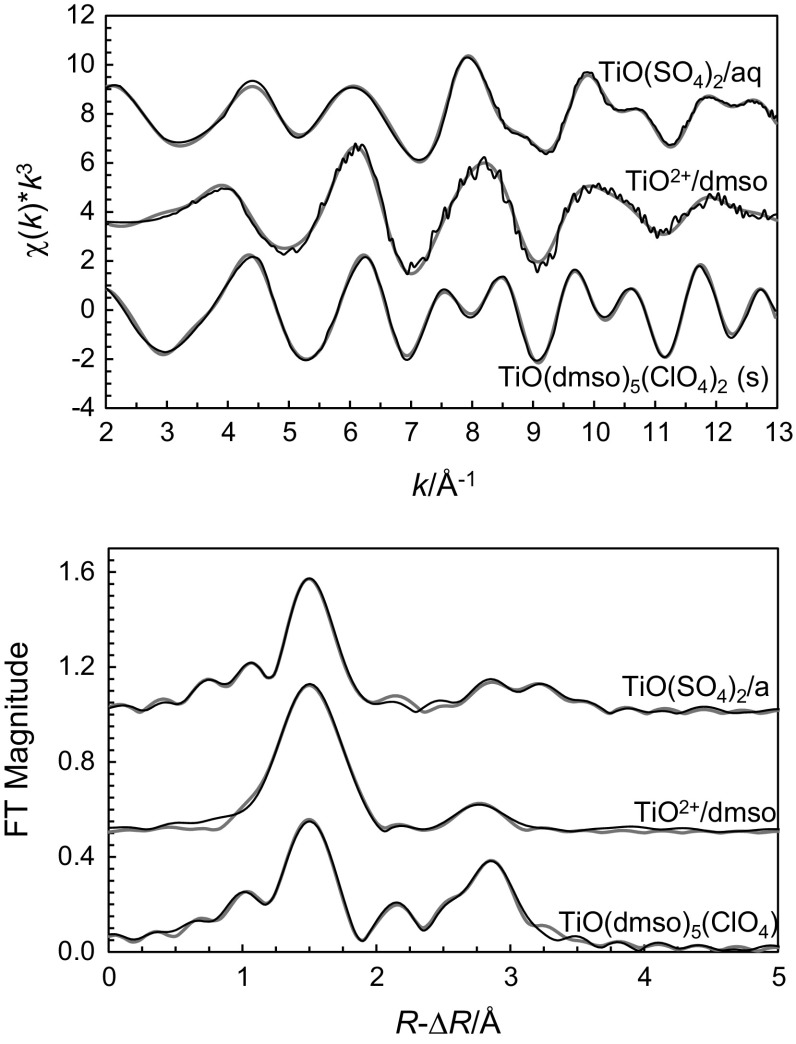
The experimental (*upper*) and the fitted Fourier-transform (*lower*) of the *k* ^3^-weighted EXAFS data of the hydrated bis(hydrogen)sulfatotitanyl(IV) complex, and the dimethylsulfoxide solvated titanyl ion in dimethylsulfoxide solution and in solid state as trifluoromethanesulfonate salt; experimental data, *black line*; model, *grey line*

The EXAFS study on the same aqueous solution as studied by LAXS fully supports the structural information obtained by LAXS. The structure parameters have been refined to 1.65(1) Å for the Ti=O bond, 2.036(8) Å for the four oxygens in the equatorial plane and 2.22(4) Å for the oxygen in the axial position, Table 1. The Ti–(O)–S single scattering distance was refined to 3.49(2) Å and the corresponding Ti–O–S 3-leg scattering path to 3.51(4) Å, showing that the (hydrogen)sulfate ions bind to titanium with an almost linear Ti–O–S bond angle. The fit of the EXAFS spectrum is shown in Fig. 2, and the refined structure parameters are given in Table 4.

### Dimethylsulfoxide Solvated Titanyl(IV) Ion

**Fig. 3:**
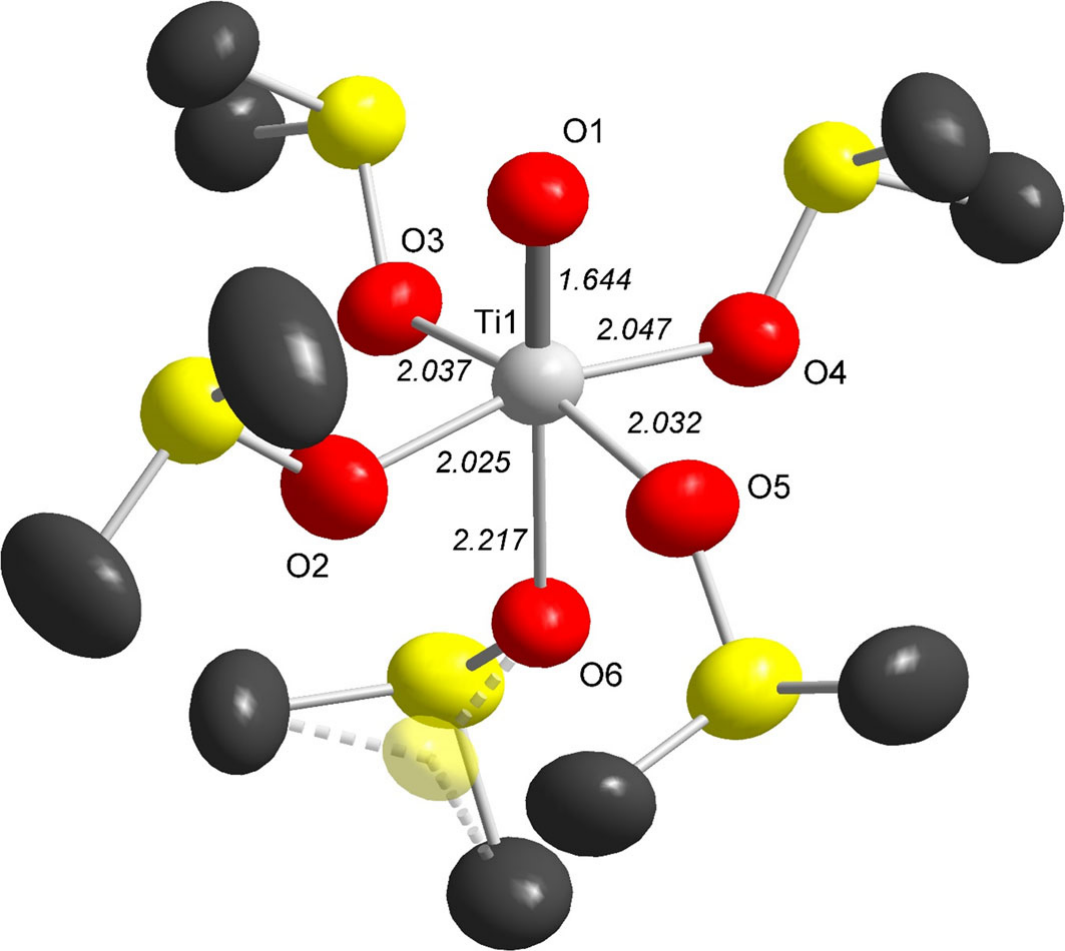
Structure of [TiO(dmso)_5_]^2+^ unit in **1**. Thermal ellipsoids of 50%. Hydrogen atoms have been removed for clarity. The lower occupancy sulfur atom on oxygen O6 has been *shaded*, as is the case with its *dashed* bonds

The *O*-donor solvent DMSO solvates titanyl(IV) well, e.g., as seen in the fact that the only reported structures of solvated titanyl(IV) ions are two determinations of the chloride salt [10, 11]. In this study, the structures of the pentakis(DMSO)titanyl(IV) complex in both solution and in the solid state, as the trifluoromethanesulfonate salt, **1**, are reported. The crystal structure of **1** shows that the pentakis(DMSO)titanyl(IV) unit has an almost identical structure as the one reported for the chloride salt [10, 11] with a Ti=O bond distance of 1.644 Å, a mean Ti–O bond distance to the equatorially bound DMSO molecules of 2.035 Å, and a Ti–O bond distance to the axially bound DMSO molecule of 2.217 Å. The equatorial plane of four DMSO molecules is slightly tilted away from the titanyl double bond with a mean O=Ti–O_eq_ bond angle of 97.3°, Table 5. Selected bond distances and angles are given in Table 5, a comparison of bond distances of structures containing titanyl ions in Table 6, and the .cif file of **1** in supplementary Table S1. The structure of the [TiO(OS(CH_3_)_2_)_5_]^2+^ unit is shown in Fig. 3, and its position in the unit cell in supplementary Fig. S1. The structure parameters obtained crystallographically are fully supported by the EXAFS study with Ti=O, Ti–O_eq_ and Ti–O_ax_ bond distances of 1.648(8), 2.040(5) and 2.23(2) Å, respectively, Table 4 and Fig. 2.

**Table 5:** Selected bond lengths (Å) and angles (°) in **1**

Bond distances		Bond angles			
Ti=O1	1.644(4)	O1=Ti–O2	100.1(2)	O2–Ti–O3	88.8(2)
Ti–O2	2.025(4)	O1=Ti–O3	95.1(2)	O2–Ti–O4	164.3(2)
Ti–O5	2.032(4)	O1=Ti–O4	95.6(2)	O2–Ti–O5	88.3(2)
Ti–O3	2.037(4)	O1=Ti–O5	98.3(2)	O2–Ti–O6	82.2(2)
Ti–O4	2.047(4)	O1=Ti–O6	176.9(2)	O3–Ti–O4	91.1(2)
Ti–O6	2.217(4)			O3–Ti–O5	166.6(2)
				O3–Ti–O6	82.8(2)
				O4–Ti–O5	88.1(2)
				O4–Ti–O6	82.2(2)
				O5–Ti–O6	83.8(2)
		Mean O1=Ti–O_eq_	97.3	Mean O6–Ti–O_eq_	82.8

**Table 6:** Summary of Ti=O and Ti–O bond distances (Å) in isolated titanyl(IV) complexes both in solid state and solution

Titanyl(IV) Complex	*d*(Ti=O)	*d*(Ti–O_eq_)	*d*(Ti–O_ax_)	Ref.	Method
Solid state					
Ba_2_[TiO(Si_2_O_7_)_2_] (s)	1.634	4*2.001	–	[7]	XRD
Sr_2_[TiO(PO_4_)_2_]	1.747	2.001/2.010/2.022/2.032	2.042	[8]	XRD
[TiO(DMSO)_5_]Cl_2_(s)	1.650	2.017/2.021/2.040/2.049	2.223	[10]	XRD
[TiO(DMSO)_5_]Cl_2_(s)	1.648	2.025/2.028/2.040/2.047	2.234	[11]	XRD
[TiO(DMSO)_5_](CF_3_SO_3_)_2_(s)	1.644	2.025/2.032/2.037/2.047	2.217	*	XRD
[TiO(DMSO)_5_](CF_3_SO_3_)_2_(s)	1.648	4*2.040	2.23	*	EXAFS
(C(NH_2_)_3_)_4_[TiO(CO_3_)_3_]·2H_2_O	1.680	2.071/2.083/2.088/2.138/2.158	2.230	[9]	XRD
Solution					
[TiO(DMSO)_5_]^2+^/DMSO	1.646	4*2.035	2.22	*	EXAFS
[TiO(SO_4_)_2_]^2−^/aq	1.642	4*2.025	2.21	*	LAXS
[TiO(SO_4_)_2_]^2−^/aq	1.646	4*2.036	2.22	*	EXAFS

* Denotes this work

An EXAFS study of the DMSO solvated titanyl(IV) ion in DMSO solution shows that the structure in the solid state is maintained in DMSO solution with refined Ti=O, Ti–O_eq_ and Ti–O_ax_ bond distances of 1.65(2), 2.035(10) and 2.22(4) Å, respectively, Table 4 and Fig. 2. However, the Debye–Waller coefficients are significantly larger for the complex in solution than in the solid state, indicating a larger bond distance distribution of the complex in solution. This is clearly seen in a comparison of the EXAFS spectra collected in solution and solid state with the same features in the EXAFS spectra but smoothed out in the solution spectrum, Fig. 2. The structure parameters are summarized in Table 4, and the fit of the EXAFS data are shown in Fig. 2.

## Conclusions

The titanyl(IV) ion must be stabilized through complexation or strong solvation, by, e.g., (hydrogen)sulfate ion and DMSO, respectively, to be able to exist as individual units as described in this paper. Without stabilization in aqueous systems, it will immediately be hydrolyzed to titanium(IV) oxide. The structure of the titanyl(IV) ion consists of a short Ti=O bond at ca. 1.64 Å, four solvent molecules or ligands at ca. 2.02 Å and with a O=Ti–O bond angle of 95°–100°, and a weakly bound ligand *trans* to the Ti=O bond with a Ti–O bond distance of ca. 2.22 Å. For a comparison of the titanyl(IV) ion being stabilized by ligands such as sulfate, phosphate, silicate, and DMSO, see Table 6.

## Electronic supplementary material

Below is the link to the electronic supplementary material.

Supplementary material 1 (PDF 260 kb)
