# Supplementary material for: Structure of a Hydrated Sulfonatotitanyl(IV) Complex in Aqueous Solution and the Dimethylsulfoxide Solvated Titanyl(IV) Ion in Solution and Solid State
Source: J Solution Chem. 2017 Jan 27;46(2):476–87. doi: 10.1007/s10953-017-0581-3 (PMC5321709; doi:10.1007/s10953-017-0581-3)

Structure of a hydrated sulfonatotitanyl(IV) complex in aqueous solution and the dimethylsulfoxide solvated titanyl(IV) ion in solution and solid state

Daniel Lundberg and Ingmar Persson*

Department of Chemistry and Biotechnology, Swedish University of Agricultural Sciences, P.O. Box 7015, SE-750 07 Uppsala, Sweden.

Electronic supplementary information

Table S1. Cif file for $[TiO(OS(CH_3)_2)_5](CF_3SO_3)_2$.

```
data_tidmso2m
_publ_requested_journal          Dalton Transactions
_publ_contact_author_name        'Daniel Lundberg'
_publ_contact_author_address
;Department of Chemistry and Biotechnology
Uppsala BioCenter
Swedish University of Agricultural Sciences
P.O. Box 7015
SE-750 07 Uppsala
SWEDEN
;
_publ_contact_author_email       daniel.lundberg@slu.se
_publ_contact_author_phone       +46-18-671549
loop_
_publ_author_name
_publ_author_address
'Lundberg, Daniel'
;Department of Chemistry and Biotechnology
Uppsala BioCenter
Swedish University of Agricultural Sciences
P.O. Box 7015
SE-750 07 Uppsala
SWEDEN
;
'Persson, Ingmar'
;Department of Chemistry and Biotechnology
Uppsala BioCenter
Swedish University of Agricultural Sciences
P.O. Box 7015
SE-750 07 Uppsala
SWEDEN
;

_audit_creation_method           SHELXL-2014/7
_chemical_name_systematic
'pentakis(dimethylsulfoxide)oxotitanium(IV) trifluoromethanesulfonate'
_chemical_name_common            'pentakis(dmso)oxotitanium(IV) triflate'
_chemical_formula_moiety         'Ti O6 S5 C10 H30, 2 (C F3 S O3)'
_chemical_formula_sum            'C12 H30 F6 O12 S7 Ti'
_chemical_compound_source        synthesized
_exptl_crystal_recrystallization_method 'crystallized from dmso'
_chemical_melting_point          ?

_exptl_crystal_description       block
_exptl_crystal_colour            colourless
_diffrn_ambient_temperature      295(2)
_chemical_formula_weight         752.68

loop_
_atom_type_symbol
_atom_type_description
_atom_type_scat_dispersion_real
_atom_type_scat_dispersion_imag
```

_atom_type_scat_source
C C 0.0033 0.0016 'International Tables Vol C Tables 4.2.6.8 and 6.1.1.4'
H H 0.0000 0.0000 'International Tables Vol C Tables 4.2.6.8 and 6.1.1.4'
O O 0.0106 0.0060 'International Tables Vol C Tables 4.2.6.8 and 6.1.1.4'
F F 0.0171 0.0103 'International Tables Vol C Tables 4.2.6.8 and 6.1.1.4'
S S 0.1246 0.1234 'International Tables Vol C Tables 4.2.6.8 and 6.1.1.4'
Ti Ti 0.2776 0.4457 'International Tables Vol C Tables 4.2.6.8 and 6.1.1.4'

_space_group_crystal_system triclinic
_space_group_IT_number 2
_space_group_name_H-M_alt 'P -1'
_space_group_name_Hall '-P 1'

_shelx_space_group_comment
;
The symmetry employed for this shelxl refinement is uniquely defined
by the following loop, which should always be used as a source of
symmetry information in preference to the above space-group names.
They are only intended as comments.
;

loop_
_space_group_symop_operation_xyz
'x, y, z'
'-x, -y, -z'

_cell_length_a 10.198(5)
_cell_length_b 13.495(6)
_cell_length_c 13.797(6)
_cell_angle_alpha 99.002(9)
_cell_angle_beta 110.814(8)
_cell_angle_gamma 110.231(8)
_cell_volume 1578.5(13)
_cell_formula_units_Z 2
_cell_measurement_temperature 295(2)
_cell_measurement_reflns_used 4797
_cell_measurement_theta_min 2.93
_cell_measurement_theta_max 24.93
_exptl_crystal_density_meas ?
_exptl_crystal_density_method 'not measured'
_exptl_crystal_density_diffrn 1.584
_exptl_crystal_F_000 772
_exptl_transmission_factor_min ?
_exptl_transmission_factor_max ?
_exptl_crystal_size_max 0.60
_exptl_crystal_size_mid 0.20
_exptl_crystal_size_min 0.15
_exptl_absorpt_coefficient_mu 0.816
_shelx_estimated_absorpt_T_min ?
_shelx_estimated_absorpt_T_max ?
_exptl_absorpt_correction_type multi-scan
_exptl_absorpt_correction_T_min 0.054932
_exptl_absorpt_correction_T_max 0.121029
_exptl_absorpt_process_details 'SADABS, Bruker (2004)'
_diffrn_radiation_probe x-ray

_diffrn_radiation_type MoK\a
_diffrn_radiation_wavelength 0.71073
_diffrn_source 'fine-focus sealed tube'
_diffrn_source_type 'Bruker SMART'
_diffrn_measurement_device_type 'CCD area detector'
_diffrn_measurement_method '\f and\w scans'
_diffrn_detector_area_resol_mean ?
_diffrn_reflns_number 5575
_diffrn_reflns_av_unetI/netI 0.0284
_diffrn_reflns_av_R_equivalents 0.0314
_diffrn_reflns_limit_h_min -11
_diffrn_reflns_limit_h_max 11
_diffrn_reflns_limit_k_min -14
_diffrn_reflns_limit_k_max 14
_diffrn_reflns_limit_l_min -7
_diffrn_reflns_limit_l_max 15
_diffrn_reflns_theta_min 1.666
_diffrn_reflns_theta_max 22.984
_diffrn_reflns_theta_full 22.984
_diffrn_measured_fraction_theta_max 0.950
_diffrn_measured_fraction_theta_full 0.950
_diffrn_reflns_Laue_measured_fraction_max 0.950
_diffrn_reflns_Laue_measured_fraction_full 0.950
_diffrn_reflns_point_group_measured_fraction_max 0.950
_diffrn_reflns_point_group_measured_fraction_full 0.950
_reflns_number_total 4170
_reflns_number_gt 3327
_reflns_threshold_expression 'I > 2\s(I)'
_reflns_Friedel_coverage 0.000
_reflns_Friedel_fraction_max .
_reflns_Friedel_fraction_full .

_reflns_special_details
;
Reflections were merged by SHELXL according to the crystal
class for the calculation of statistics and refinement.

_reflns_Friedel_fraction is defined as the number of unique
Friedel pairs measured divided by the number that would be
possible theoretically, ignoring centric projections and
systematic absences.
;

_computing_data_collection 'Bruker SMART'
_computing_cell_refinement 'Bruker SMART'
_computing_data_reduction 'Bruker SHELXTL'
_computing_structure_solution 'Bruker SHELXTL'
_computing_structure_refinement 'SHELXL-2014/7 (Sheldrick, 2014)'
_computing_molecular_graphics 'Diamond 2.0'
_computing_publication_material 'Diamond 2.0'
_refine_special_details ?
_refine_ls_structure_factor_coef Fsqd
_refine_ls_matrix_type full
_refine_ls_weighting_scheme calc
_refine_ls_weighting_details

'w=1/[\s^2^(Fo^2^)+(0.1597P)^2^+0.5380P] where P=(Fo^2^+2Fc^2^)/3'
_atom_sites_solution_primary ?
_atom_sites_solution_secondary ?
_atom_sites_solution_hydrogens geom
_refine_ls_hydrogen_treatment constr
_refine_ls_extinction_method none
_refine_ls_extinction_coef .
_refine_ls_number_reflns 4170
_refine_ls_number_parameters 410
_refine_ls_number_restraints 24
_refine_ls_R_factor_all 0.0856
_refine_ls_R_factor_gt 0.0721
_refine_ls_wR_factor_ref 0.2216
_refine_ls_wR_factor_gt 0.2031
_refine_ls_goodness_of_fit_ref 1.092
_refine_ls_restrained_S_all 1.094
_refine_ls_shift/su_max 0.000
_refine_ls_shift/su_mean 0.000

loop_
_atom_site_label
_atom_site_type_symbol
_atom_site_fract_x
_atom_site_fract_y
_atom_site_fract_z
_atom_site_U_iso_or_equiv
_atom_site_adp_type
_atom_site_occupancy
_atom_site_site_symmetry_order
_atom_site_calc_flag
_atom_site_refinement_flags_posn
_atom_site_refinement_flags_adp
_atom_site_refinement_flags_occupancy
_atom_site_disorder_assembly
_atom_site_disorder_group
Ti1 Ti 0.28116(11) 0.77445(7) 0.29752(8) 0.0581(4) Uani 1 1 d . . . . .
O1 O 0.4131(5) 0.9015(3) 0.3752(3) 0.0702(10) Uani 1 1 d . . . . .
O2 O 0.2718(5) 0.7013(3) 0.4139(3) 0.0791(11) Uani 1 1 d . . . . .
S2 S 0.3365(2) 0.75125(14) 0.53820(14) 0.0818(5) Uani 1 1 d . . . . .
C21 C 0.2865(12) 0.6326(8) 0.5799(8) 0.138(4) Uani 1 1 d . . . . .
H21A H 0.3224 0.6552 0.6578 0.206 Uiso 1 1 calc R U . . .
H21B H 0.1754 0.5899 0.5444 0.206 Uiso 1 1 calc R U . . .
H21C H 0.3345 0.5880 0.5604 0.206 Uiso 1 1 calc R U . . .
C22 C 0.5347(10) 0.8025(10) 0.5930(8) 0.157(5) Uani 1 1 d . . . . .
H22A H 0.5785 0.8344 0.6709 0.235 Uiso 1 1 calc R U . . .
H22B H 0.5625 0.7432 0.5762 0.235 Uiso 1 1 calc R U . . .
H22C H 0.5744 0.8587 0.5627 0.235 Uiso 1 1 calc R U . . .
O3 O 0.0964(4) 0.8028(3) 0.2950(3) 0.0716(10) Uani 1 1 d . . . . .
S3 S 0.10832(18) 0.92142(12) 0.33001(12) 0.0695(5) Uani 1 1 d . . . . .
C31 C 0.1986(8) 0.9675(6) 0.4756(5) 0.0870(19) Uani 1 1 d . . . . .
H31A H 0.2092 1.0413 0.5022 0.131 Uiso 1 1 calc R U . . .
H31B H 0.1357 0.9178 0.5011 0.131 Uiso 1 1 calc R U . . .
H31C H 0.2998 0.9682 0.5020 0.131 Uiso 1 1 calc R U . . .
C32 C -0.0838(8) 0.8947(6) 0.3064(6) 0.0848(18) Uani 1 1 d . . . . .
H32A H -0.0898 0.9639 0.3257 0.127 Uiso 1 1 calc R U . . .

H32B H -0.1528 0.8545 0.2304 0.127 Uiso 1 1 calc R U . . .
H32C H -0.1136 0.8510 0.3501 0.127 Uiso 1 1 calc R U . . .
O4 O 0.2497(5) 0.8050(3) 0.1521(3) 0.0705(10) Uani 1 1 d . . . . .
S4 S 0.32109(18) 0.92260(12) 0.14652(12) 0.0692(5) Uani 1 1 d . . . . .
C41 C 0.2611(10) 0.8970(6) 0.0047(6) 0.099(2) Uani 1 1 d . . . . .
H41A H 0.2998 0.9663 -0.0092 0.149 Uiso 1 1 calc R U . . .
H41B H 0.3014 0.8493 -0.0208 0.149 Uiso 1 1 calc R U . . .
H41C H 0.1493 0.8615 -0.0331 0.149 Uiso 1 1 calc R U . . .
C42 C 0.5208(8) 0.9600(7) 0.1889(6) 0.100(2) Uani 1 1 d . . . . .
H42A H 0.5709 1.0338 0.1867 0.150 Uiso 1 1 calc R U . . .
H42B H 0.5669 0.9583 0.2622 0.150 Uiso 1 1 calc R U . . .
H42C H 0.5336 0.9084 0.1408 0.150 Uiso 1 1 calc R U . . .
O5 O 0.4264(5) 0.7094(3) 0.2790(4) 0.0768(11) Uani 1 1 d . . . . .
S5 S 0.39056(19) 0.59353(14) 0.21376(14) 0.0803(5) Uani 1 1 d . . . . .
C51 C 0.5686(10) 0.6139(7) 0.2097(8) 0.115(3) Uani 1 1 d . . . . .
H51A H 0.5592 0.5448 0.1697 0.173 Uiso 1 1 calc R U . . .
H51B H 0.5942 0.6685 0.1742 0.173 Uiso 1 1 calc R U . . .
H51C H 0.6495 0.6394 0.2829 0.173 Uiso 1 1 calc R U . . .
C52 C 0.3939(11) 0.5162(6) 0.3054(7) 0.108(2) Uani 1 1 d . . . . .
H52A H 0.3720 0.4416 0.2690 0.162 Uiso 1 1 calc R U . . .
H52B H 0.4950 0.5505 0.3669 0.162 Uiso 1 1 calc R U . . .
H52C H 0.3166 0.5142 0.3303 0.162 Uiso 1 1 calc R U . . .
O6 O 0.0958(4) 0.6071(3) 0.1872(3) 0.0671(10) Uani 1 1 d . . . . .
S6 S -0.0761(2) 0.5540(3) 0.15284(16) 0.0768(10) Uani 0.886(12) 1 d . . P A 1
S6B S -0.034(3) 0.5074(16) 0.181(2) 0.093(10) Uani 0.114(12) 1 d . . P A 2
C61 C -0.1380(10) 0.4152(6) 0.0713(7) 0.107(2) Uani 1 1 d . . . . .
H61A H -0.2483 0.3741 0.0452 0.161 Uiso 1 1 calc R U . A 1
H61B H -0.1139 0.4174 0.0103 0.161 Uiso 1 1 calc R U . A 1
H61C H -0.0849 0.3796 0.1148 0.161 Uiso 1 1 calc R U . A 1
C62 C -0.1000(9) 0.5265(7) 0.2664(6) 0.100(2) Uani 1 1 d . . . . .
H62A H -0.2090 0.4922 0.2480 0.150 Uiso 1 1 calc R U . A 1
H62B H -0.0549 0.4771 0.2881 0.150 Uiso 1 1 calc R U . A 1
H62C H -0.0492 0.5949 0.3256 0.150 Uiso 1 1 calc R U . A 1
S20 S 0.4261(2) 0.24379(14) 0.07376(14) 0.0836(5) Uani 1 1 d D . . . .
O21 O 0.450(4) 0.182(2) 0.144(2) 0.176(14) Uani 0.46(3) 1 d D . P B 1
O22 O 0.508(4) 0.3571(13) 0.122(3) 0.171(16) Uani 0.46(3) 1 d D . P B 1
O23 O 0.418(4) 0.202(3) -0.0268(15) 0.187(14) Uani 0.46(3) 1 d D . P B 1
O21B O 0.426(4) 0.254(3) -0.0244(17) 0.223(14) Uani 0.54(3) 1 d D . P B 2
O22B O 0.499(3) 0.3447(13) 0.1566(19) 0.128(8) Uani 0.54(3) 1 d D . P B 2
O23B O 0.472(2) 0.1639(14) 0.107(2) 0.161(11) Uani 0.54(3) 1 d D . P B 2
C20 C 0.2217(12) 0.2042(9) 0.0388(8) 0.131(3) Uani 1 1 d D . . . .
F21 F 0.138(3) 0.1023(13) 0.005(3) 0.22(2) Uani 0.46(3) 1 d D . P B 1
F22 F 0.212(3) 0.253(3) 0.123(2) 0.30(2) Uani 0.46(3) 1 d D . P B 1
F23 F 0.179(4) 0.249(3) -0.035(2) 0.253(18) Uani 0.46(3) 1 d D . P B 1
F21B F 0.197(2) 0.171(3) 0.1181(13) 0.229(15) Uani 0.54(3) 1 d D . P B 2
F22B F 0.176(2) 0.2757(17) 0.021(3) 0.246(19) Uani 0.54(3) 1 d D . P B 2
F23B F 0.142(3) 0.1155(17) -0.0450(16) 0.196(12) Uani 0.54(3) 1 d D . P B 2
S10 S 0.8772(3) 0.71563(18) 0.55026(16) 0.0980(6) Uani 1 1 d . . . . .
O11 O 0.7334(8) 0.6367(6) 0.5409(7) 0.163(3) Uani 1 1 d . . . . .
O12 O 0.9481(12) 0.6649(10) 0.5095(9) 0.238(6) Uani 1 1 d . . . . .
O13 O 0.8626(10) 0.8063(7) 0.5181(7) 0.177(3) Uani 1 1 d . . . . .
C10 C 0.9929(14) 0.7738(8) 0.6929(9) 0.130(3) Uani 1 1 d . . . . .
F11 F 1.0006(14) 0.7097(8) 0.7443(7) 0.265(6) Uani 1 1 d . . . . .
F12 F 0.9456(13) 0.8373(8) 0.7403(7) 0.223(4) Uani 1 1 d . . . . .
F13 F 1.1330(8) 0.8453(9) 0.7113(7) 0.230(4) Uani 1 1 d . . . . .

```
loop_
_atom_site_aniso_label
_atom_site_aniso_U_11
_atom_site_aniso_U_22
_atom_site_aniso_U_33
_atom_site_aniso_U_23
_atom_site_aniso_U_13
_atom_site_aniso_U_12
Ti1 0.0634(6) 0.0520(6) 0.0608(6) 0.0157(4) 0.0292(5) 0.0262(5)
O1 0.073(2) 0.061(2) 0.069(2) 0.0152(18) 0.0306(19) 0.0231(19)
O2 0.093(3) 0.076(3) 0.067(2) 0.029(2) 0.032(2) 0.036(2)
S2 0.0961(12) 0.0812(11) 0.0774(10) 0.0288(8) 0.0413(9) 0.0443(9)
C21 0.148(9) 0.135(8) 0.105(6) 0.068(6) 0.041(6) 0.038(6)
C22 0.088(6) 0.206(12) 0.108(7) 0.052(7) 0.022(5) 0.012(7)
O3 0.072(2) 0.064(2) 0.084(2) 0.0146(19) 0.039(2) 0.0337(19)
S3 0.0793(10) 0.0625(9) 0.0814(10) 0.0233(7) 0.0448(8) 0.0368(8)
C31 0.093(5) 0.081(4) 0.081(4) 0.008(3) 0.039(4) 0.038(4)
C32 0.084(4) 0.092(4) 0.097(5) 0.031(4) 0.046(4) 0.052(4)
O4 0.081(2) 0.061(2) 0.062(2) 0.0160(17) 0.0300(19) 0.0254(19)
S4 0.0833(10) 0.0582(8) 0.0692(9) 0.0195(6) 0.0398(8) 0.0282(7)
C41 0.126(6) 0.085(5) 0.081(4) 0.029(4) 0.047(4) 0.037(4)
C42 0.083(5) 0.108(5) 0.099(5) 0.036(4) 0.047(4) 0.022(4)
O5 0.072(2) 0.068(2) 0.098(3) 0.021(2) 0.040(2) 0.037(2)
S5 0.0796(10) 0.0745(10) 0.0890(11) 0.0171(8) 0.0359(9) 0.0413(8)
C51 0.104(6) 0.102(6) 0.161(8) 0.031(5) 0.078(6) 0.054(5)
C52 0.137(7) 0.080(5) 0.117(6) 0.036(4) 0.059(5) 0.052(5)
O6 0.062(2) 0.055(2) 0.076(2) 0.0114(17) 0.0340(19) 0.0165(18)
S6 0.0698(12) 0.0641(19) 0.0911(13) 0.0246(10) 0.0302(10) 0.0289(10)
S6B 0.134(17) 0.052(9) 0.135(16) 0.039(10) 0.101(14) 0.037(11)
C61 0.104(6) 0.083(5) 0.101(5) 0.009(4) 0.041(4) 0.016(4)
C62 0.090(5) 0.103(5) 0.095(5) 0.015(4) 0.056(4) 0.020(4)
S20 0.0893(12) 0.0722(11) 0.0892(12) 0.0201(9) 0.0430(9) 0.0337(9)
O21 0.22(3) 0.17(2) 0.150(16) 0.093(15) 0.048(16) 0.117(18)
O22 0.16(2) 0.067(11) 0.30(4) 0.053(16) 0.14(3) 0.030(11)
O23 0.18(2) 0.27(3) 0.127(17) -0.022(17) 0.075(14) 0.14(2)
O21B 0.20(2) 0.37(4) 0.16(2) 0.12(2) 0.134(19) 0.12(3)
O22B 0.099(12) 0.092(11) 0.134(11) -0.019(9) 0.014(9) 0.040(10)
O23B 0.103(10) 0.078(8) 0.28(3) 0.064(12) 0.052(14) 0.049(8)
C20 0.112(7) 0.158(10) 0.107(7) 0.023(7) 0.055(6) 0.042(8)
F21 0.101(16) 0.119(15) 0.39(6) 0.06(2) 0.10(3) 0.001(11)
F22 0.24(3) 0.21(3) 0.40(4) -0.08(3) 0.27(3) -0.003(19)
F23 0.29(3) 0.34(4) 0.184(19) 0.061(19) 0.040(17) 0.27(3)
F21B 0.128(12) 0.36(4) 0.144(14) 0.085(18) 0.082(11) 0.024(19)
F22B 0.131(13) 0.198(17) 0.43(5) 0.09(3) 0.10(2) 0.122(14)
F23B 0.103(14) 0.21(2) 0.131(12) 0.028(12) -0.006(9) -0.015(12)
S10 0.1011(14) 0.1042(14) 0.0851(12) 0.0278(10) 0.0413(10) 0.0414(12)
O11 0.109(5) 0.129(5) 0.181(7) 0.029(5) 0.047(5) 0.004(4)
O12 0.186(8) 0.279(12) 0.200(9) -0.063(8) 0.087(7) 0.106(8)
O13 0.192(7) 0.164(7) 0.169(7) 0.116(6) 0.052(6) 0.073(6)
C10 0.133(8) 0.104(6) 0.129(8) 0.037(6) 0.027(6) 0.056(6)
F11 0.352(13) 0.183(7) 0.151(6) 0.102(6) -0.001(7) 0.095(8)
F12 0.292(11) 0.221(8) 0.174(6) 0.008(6) 0.137(7) 0.118(8)
F13 0.099(5) 0.252(9) 0.200(8) 0.012(7) 0.010(4) 0.009(5)
```

_geom_special_details
;
 All esds (except the esd in the dihedral angle between two l.s. planes)
 are estimated using the full covariance matrix. The cell esds are taken
 into account individually in the estimation of esds in distances, angles
 and torsion angles; correlations between esds in cell parameters are only
 used when they are defined by crystal symmetry. An approximate (isotropic)
 treatment of cell esds is used for estimating esds involving l.s. planes.
;

loop_
_geom_bond_atom_site_label_1
_geom_bond_atom_site_label_2
_geom_bond_distance
_geom_bond_site_symmetry_2
_geom_bond_publ_flag
Ti1 O1 1.644(4) . ?
Ti1 O2 2.025(4) . ?
Ti1 O5 2.032(4) . ?
Ti1 O3 2.037(4) . ?
Ti1 O4 2.047(4) . ?
Ti1 O6 2.217(4) . ?
O2 S2 1.536(4) . ?
S2 C22 1.708(9) . ?
S2 C21 1.757(9) . ?
O3 S3 1.547(4) . ?
S3 C32 1.760(7) . ?
S3 C31 1.780(7) . ?
O4 S4 1.528(4) . ?
S4 C42 1.759(7) . ?
S4 C41 1.764(7) . ?
O5 S5 1.523(4) . ?
S5 C52 1.760(8) . ?
S5 C51 1.763(8) . ?
O6 S6B 1.491(17) . ?
O6 S6 1.499(4) . ?
S6 C62 1.744(8) . ?
S6 C61 1.786(8) . ?
S6B C61 1.52(2) . ?
S6B C62 1.579(16) . ?
S20 O22 1.371(16) . ?
S20 O23 1.377(13) . ?
S20 O21B 1.381(14) . ?
S20 O22B 1.386(12) . ?
S20 O21 1.387(14) . ?
S20 O23B 1.397(14) . ?
S20 C20 1.816(10) . ?
C20 F22B 1.230(15) . ?
C20 F21 1.240(16) . ?
C20 F23 1.286(18) . ?
C20 F23B 1.286(16) . ?
C20 F22 1.294(15) . ?
C20 F21B 1.316(15) . ?
S10 O12 1.348(7) . ?
S10 O13 1.402(7) . ?

S10 O11 1.430(7) . ?
S10 C10 1.763(11) . ?
C10 F11 1.207(11) . ?
C10 F12 1.314(12) . ?
C10 F13 1.323(13) . ?

loop_
_geom_angle_atom_site_label_1
_geom_angle_atom_site_label_2
_geom_angle_atom_site_label_3
_geom_angle
_geom_angle_site_symmetry_1
_geom_angle_site_symmetry_3
_geom_angle_publ_flag
O1 Ti1 O2 100.05(19) . . ?
O1 Ti1 O5 98.28(18) . . ?
O2 Ti1 O5 88.34(18) . . ?
O1 Ti1 O3 95.14(18) . . ?
O2 Ti1 O3 88.78(18) . . ?
O5 Ti1 O3 166.57(16) . . ?
O1 Ti1 O4 95.58(18) . . ?
O2 Ti1 O4 164.32(17) . . ?
O5 Ti1 O4 88.13(17) . . ?
O3 Ti1 O4 91.13(17) . . ?
O1 Ti1 O6 176.92(18) . . ?
O2 Ti1 O6 82.23(17) . . ?
O5 Ti1 O6 83.81(15) . . ?
O3 Ti1 O6 82.80(15) . . ?
O4 Ti1 O6 82.20(15) . . ?
S2 O2 Ti1 131.2(3) . . ?
O2 S2 C22 107.6(4) . . ?
O2 S2 C21 102.9(4) . . ?
C22 S2 C21 101.7(5) . . ?
S3 O3 Ti1 122.6(2) . . ?
O3 S3 C32 102.4(3) . . ?
O3 S3 C31 105.7(3) . . ?
C32 S3 C31 99.1(3) . . ?
S4 O4 Ti1 122.1(2) . . ?
O4 S4 C42 106.4(3) . . ?
O4 S4 C41 101.8(3) . . ?
C42 S4 C41 99.0(4) . . ?
S5 O5 Ti1 130.4(2) . . ?
O5 S5 C52 105.3(3) . . ?
O5 S5 C51 102.3(3) . . ?
C52 S5 C51 99.7(5) . . ?
S6B O6 Ti1 142.8(8) . . ?
S6 O6 Ti1 131.7(2) . . ?
O6 S6 C62 106.6(3) . . ?
O6 S6 C61 103.3(3) . . ?
C62 S6 C61 99.3(4) . . ?
O6 S6B C61 118.0(13) . . ?
O6 S6B C62 116.0(11) . . ?
C61 S6B C62 120.3(14) . . ?
O22 S20 O23 115.9(15) . . ?
O21B S20 O22B 114.0(13) . . ?

O22 S20 O21 115.5(15) . . ?
O23 S20 O21 114.9(13) . . ?
O21B S20 O23B 114.4(13) . . ?
O22B S20 O23B 113.0(11) . . ?
O22 S20 C20 109.1(17) . . ?
O23 S20 C20 101.1(15) . . ?
O21B S20 C20 99.8(14) . . ?
O22B S20 C20 102.3(12) . . ?
O21 S20 C20 97.1(17) . . ?
O23B S20 C20 111.8(10) . . ?
F21 C20 F23 111.1(17) . . ?
F22B C20 F23B 110.3(15) . . ?
F21 C20 F22 111.8(16) . . ?
F23 C20 F22 106.3(16) . . ?
F22B C20 F21B 111.5(14) . . ?
F23B C20 F21B 104.3(14) . . ?
F22B C20 S20 116.0(12) . . ?
F21 C20 S20 114.8(16) . . ?
F23 C20 S20 103.9(15) . . ?
F23B C20 S20 107.3(14) . . ?
F22 C20 S20 108.3(14) . . ?
F21B C20 S20 106.7(11) . . ?
O12 S10 O13 118.2(8) . . ?
O12 S10 O11 111.5(7) . . ?
O13 S10 O11 111.9(6) . . ?
O12 S10 C10 107.1(6) . . ?
O13 S10 C10 103.5(5) . . ?
O11 S10 C10 102.9(6) . . ?
F11 C10 F12 104.2(12) . . ?
F11 C10 F13 111.4(11) . . ?
F12 C10 F13 102.6(10) . . ?
F11 C10 S10 117.0(8) . . ?
F12 C10 S10 112.6(8) . . ?
F13 C10 S10 108.1(9) . . ?

_refine_diff_density_max 0.612
_refine_diff_density_min -0.795
_refine_diff_density_rms 0.096

_shelx_res_file
;

tidmso2m.res created by SHELXL-2014/7

TITL tidmso2m in P-1
CELL 0.71073 10.1976 13.4949 13.7966 99.002 110.814 110.231
ZERR 2.00 0.0048 0.0062 0.0063 0.009 0.008 0.008
LATT 1
SFAC C H O F S TI
UNIT 24 60 24 12 14 2
TEMP 22
L.S. 24
BOND
FMAP 2

```
PLAN -20
acta
shel 99 0.91
dfix 41 s20 o21 s20 o22 s20 o23
dfix 41 s20 o21b s20 o22b s20 o23b
dfix 41.633 o21 o22 o22 o23 o23 o21
dfix 41.633 o21b o22b o22b o23b o23b o21b
dfix 51 c20 f21 c20 f22 c20 f23
dfix 51 c20 f21b c20 f22b c20 f23b
dfix 51.633 f21 f22 f22 f23 f23 f21
dfix 51.633 f21b f22b f22b f23b f23b f21b
WGHT    0.159700    0.538000
FVAR       2.20121   0.88590   0.46006   1.41419   1.27364
TI1   6    0.281161    0.774455    0.297525    11.00000    0.06344    0.05196 =
        0.06084    0.01573    0.02924    0.02618
O1    3    0.413126    0.901451    0.375180    11.00000    0.07258    0.06095 =
        0.06877    0.01523    0.03060    0.02307
O2    3    0.271817    0.701276    0.413871    11.00000    0.09254    0.07579 =
        0.06724    0.02910    0.03178    0.03564
S2    5    0.336483    0.751252    0.538201    11.00000    0.09615    0.08119 =
        0.07745    0.02882    0.04127    0.04433
C21   1    0.286537    0.632622    0.579925    11.00000    0.14761    0.13526 =
        0.10540    0.06760    0.04079    0.03799
AFIX  33
H21A  2    0.322401    0.655170    0.657777    11.00000   -1.50000
H21B  2    0.175426    0.589888    0.544444    11.00000   -1.50000
H21C  2    0.334493    0.587985    0.560354    11.00000   -1.50000
AFIX   0
C22   1    0.534668    0.802506    0.593009    11.00000    0.08847    0.20604 =
        0.10799    0.05233    0.02200    0.01192
AFIX  33
H22A  2    0.578486    0.834397    0.670938    11.00000   -1.50000
H22B  2    0.562524    0.743179    0.576176    11.00000   -1.50000
H22C  2    0.574367    0.858745    0.562715    11.00000   -1.50000
AFIX   0
O3    3    0.096432    0.802760    0.295011    11.00000    0.07190    0.06359 =
        0.08402    0.01464    0.03890    0.03371
S3    5    0.108323    0.921425    0.330014    11.00000    0.07929    0.06247 =
        0.08140    0.02331    0.04479    0.03681
C31   1    0.198626    0.967492    0.475604    11.00000    0.09263    0.08086 =
        0.08146    0.00828    0.03905    0.03797
AFIX  33
H31A  2    0.209159    1.041287    0.502206    11.00000   -1.50000
H31B  2    0.135662    0.917837    0.501093    11.00000   -1.50000
H31C  2    0.299763    0.968198    0.502038    11.00000   -1.50000
AFIX   0
C32   1   -0.083816    0.894680    0.306396    11.00000    0.08415    0.09174 =
        0.09734    0.03138    0.04648    0.05160
AFIX  33
H32A  2   -0.089838    0.963928    0.325747    11.00000   -1.50000
H32B  2   -0.152827    0.854532    0.230434    11.00000   -1.50000
H32C  2   -0.113615    0.850987    0.350122    11.00000   -1.50000
AFIX   0
O4    3    0.249665    0.804952    0.152130    11.00000    0.08088    0.06098 =
        0.06168    0.01602    0.03002    0.02540
```

```
S4    5    0.321088    0.922596    0.146517    11.00000    0.08333    0.05821 =
         0.06919    0.01953    0.03984    0.02820
C41   1    0.261073    0.896986    0.004715    11.00000    0.12556    0.08491 =
         0.08052    0.02850    0.04708    0.03650
AFIX  33
H41A  2    0.299831    0.966261   -0.009203    11.00000   -1.50000
H41B  2    0.301386    0.849293   -0.020755    11.00000   -1.50000
H41C  2    0.149347    0.861470   -0.033057    11.00000   -1.50000
AFIX   0
C42   1    0.520792    0.960021    0.188868    11.00000    0.08284    0.10802 =
         0.09917    0.03572    0.04691    0.02231
AFIX  33
H42A  2    0.570921    1.033787    0.186681    11.00000   -1.50000
H42B  2    0.566864    0.958328    0.262195    11.00000   -1.50000
H42C  2    0.533576    0.908370    0.140839    11.00000   -1.50000
AFIX   0
O5    3    0.426368    0.709401    0.278959    11.00000    0.07205    0.06757 =
         0.09764    0.02066    0.04024    0.03734
S5    5    0.390559    0.593532    0.213764    11.00000    0.07955    0.07453 =
         0.08904    0.01708    0.03586    0.04126
C51   1    0.568645    0.613868    0.209686    11.00000    0.10444    0.10164 =
         0.16118    0.03118    0.07784    0.05353
AFIX  33
H51A  2    0.559213    0.544787    0.169740    11.00000   -1.50000
H51B  2    0.594197    0.668474    0.174187    11.00000   -1.50000
H51C  2    0.649480    0.639416    0.282911    11.00000   -1.50000
AFIX   0
C52   1    0.393905    0.516157    0.305399    11.00000    0.13700    0.07970 =
         0.11705    0.03599    0.05944    0.05188
AFIX  33
H52A  2    0.371978    0.441573    0.268999    11.00000   -1.50000
H52B  2    0.494973    0.550521    0.366859    11.00000   -1.50000
H52C  2    0.316590    0.514166    0.330329    11.00000   -1.50000
AFIX   0
O6    3    0.095820    0.607096    0.187240    11.00000    0.06224    0.05466 =
         0.07590    0.01144    0.03404    0.01646
part 1
S6    5   -0.076120    0.553980    0.152844    21.00000    0.06984    0.06408 =
         0.09112    0.02463    0.03023    0.02891
part 2
S6B   5   -0.034024    0.507379    0.181038   -21.00000    0.13371    0.05179 =
         0.13471    0.03851    0.10097    0.03734
part 0
C61   1   -0.137968    0.415225    0.071347    11.00000    0.10364    0.08277 =
         0.10130    0.00891    0.04132    0.01627
AFIX  33
H61A  2   -0.248329    0.374082    0.045197    11.00000   -1.50000
H61B  2   -0.113875    0.417369    0.010251    11.00000   -1.50000
H61C  2   -0.084950    0.379628    0.114779    11.00000   -1.50000
AFIX   0
C62   1   -0.099974    0.526454    0.266385    11.00000    0.08985    0.10297 =
         0.09502    0.01522    0.05557    0.02017
AFIX  33
H62A  2   -0.209011    0.492190    0.247970    11.00000   -1.50000
H62B  2   -0.054856    0.477090    0.288086    11.00000   -1.50000
```

```
H62C  2   -0.049187    0.594929    0.325598    11.00000   -1.50000
AFIX   0
rem
rem counter ions
rem

S20   5    0.426088    0.243789    0.073760    11.00000    0.08928    0.07222 =
         0.08923    0.02006    0.04301    0.03370
part 1
O21   3    0.450055    0.181625    0.143807    31.00000    0.22254    0.16967 =
         0.14960    0.09324    0.04817    0.11688
O22   3    0.507867    0.357104    0.121536    31.00000    0.15662    0.06687 =
         0.30411    0.05293    0.13660    0.02975
O23   3    0.418159    0.201582   -0.026794    31.00000    0.18251    0.26619 =
         0.12681   -0.02203    0.07544    0.14122
part 2
O21B  3    0.426077    0.253528   -0.024392   -31.00000    0.19949    0.37071 =
         0.15762    0.12311    0.13351    0.11538
O22B  3    0.498876    0.344710    0.156560   -31.00000    0.09863    0.09198 =
         0.13365   -0.01903    0.01415    0.04017
O23B  3    0.472473    0.163908    0.107126   -31.00000    0.10313    0.07841 =
         0.28242    0.06404    0.05250    0.04867
part 0
C20   1    0.221710    0.204167    0.038782    11.00000    0.11175    0.15818 =
         0.10699    0.02266    0.05463    0.04182
part 1
F21   4    0.138499    0.102294    0.004724    31.00000    0.10115    0.11934 =
         0.38935    0.06425    0.10353    0.00092
F22   4    0.212224    0.253476    0.122631    31.00000    0.24189    0.21112 =
         0.39723   -0.07772    0.26652   -0.00252
F23   4    0.179304    0.249398   -0.034768    31.00000    0.28738    0.33908 =
         0.18405    0.06122    0.04042    0.26822
part 2
F21B  4    0.197283    0.170978    0.118144   -31.00000    0.12842    0.36175 =
         0.14367    0.08545    0.08225    0.02439
F22B  4    0.175852    0.275687    0.021206   -31.00000    0.13071    0.19845 =
         0.43205    0.08654    0.10454    0.12175
F23B  4    0.142017    0.115498   -0.044978   -31.00000    0.10300    0.21020 =
         0.13125    0.02792   -0.00576   -0.01531
part 0
S10   5    0.877218    0.715627    0.550263    11.00000    0.10106    0.10421 =
         0.08510    0.02783    0.04132    0.04144
O11   3    0.733383    0.636705    0.540942    11.00000    0.10877    0.12896 =
         0.18111    0.02930    0.04700    0.00353
O12   3    0.948124    0.664911    0.509512    11.00000    0.18648    0.27942 =
         0.19994   -0.06316    0.08710    0.10591
O13   3    0.862571    0.806308    0.518123    11.00000    0.19220    0.16417 =
         0.16930    0.11596    0.05229    0.07270
C10   1    0.992851    0.773799    0.692947    11.00000    0.13290    0.10409 =
         0.12878    0.03659    0.02662    0.05615
F11   4    1.000578    0.709655    0.744332    11.00000    0.35178    0.18344 =
         0.15139    0.10171   -0.00083    0.09500
F12   4    0.945581    0.837324    0.740256    11.00000    0.29188    0.22130 =
         0.17354    0.00769    0.13720    0.11824
F13   4    1.132961    0.845325    0.711271    11.00000    0.09854    0.25203 =
```

```
        0.19989    0.01219    0.01018    0.00919
rem
HKLF 4

REM  tidmso2m in P-1
REM R1 =  0.0721 for    3327 Fo > 4sig(Fo)  and  0.0856 for all    4170 data
REM    410 parameters refined using     24 restraints

END

WGHT      0.1571      1.1244

REM Highest difference peak  0.612,  deepest hole -0.795,  1-sigma level  0.096
Q1    1   0.3851  0.7003  0.4997  11.00000  0.05    0.61
Q2    1   1.1362  0.7530  0.7475  11.00000  0.05    0.42
Q3    1   0.9896  0.7784  0.5848  11.00000  0.05    0.38
Q4    1   0.5193  0.6322  0.2805  11.00000  0.05    0.37
Q5    1   0.9384  0.5979  0.6422  11.00000  0.05    0.33
Q6    1   0.8825  0.7373  0.7192  11.00000  0.05    0.32
Q7    1  -0.1974  0.5624  0.0832  11.00000  0.05    0.32
Q8    1   1.0186  0.6401  0.6474  11.00000  0.05    0.31
Q9    1   0.3171  0.8336  0.3786  11.00000  0.05    0.31
Q10   1   0.8659  0.6300  0.5344  11.00000  0.05    0.30
Q11   1   0.3535  1.0156  0.5179  11.00000  0.05    0.29
Q12   1   0.1983  0.7177  0.1180  11.00000  0.05    0.29
Q13   1   0.2208  0.6607  0.2590  11.00000  0.05    0.29
Q14   1   0.3294  0.8719  0.3269  11.00000  0.05    0.28
Q15   1   0.4689  0.5729  0.4046  11.00000  0.05    0.28
Q16   1   0.5000  1.0000  0.5000  10.50000  0.05    0.26
Q17   1   0.4081  0.8413  0.3535  11.00000  0.05    0.26
Q18   1  -0.2450  0.5647  0.0018  11.00000  0.05    0.26
Q19   1   0.3810  1.0179  0.1798  11.00000  0.05    0.25
Q20   1   0.2386  0.5989  0.3972  11.00000  0.05    0.25
;
_shelx_res_checksum             41709
```

Figure S1. The atoms in the asymmetric unit of the unit cell of $[TiO(OS(CH_3)_2)_5](CF_3SO_3)_2$. The alternate positions of partially occupied atoms, S6b and half of the C and F atoms of trifluoromethanesulfonate ion 2 and their bonds, have been shaded.

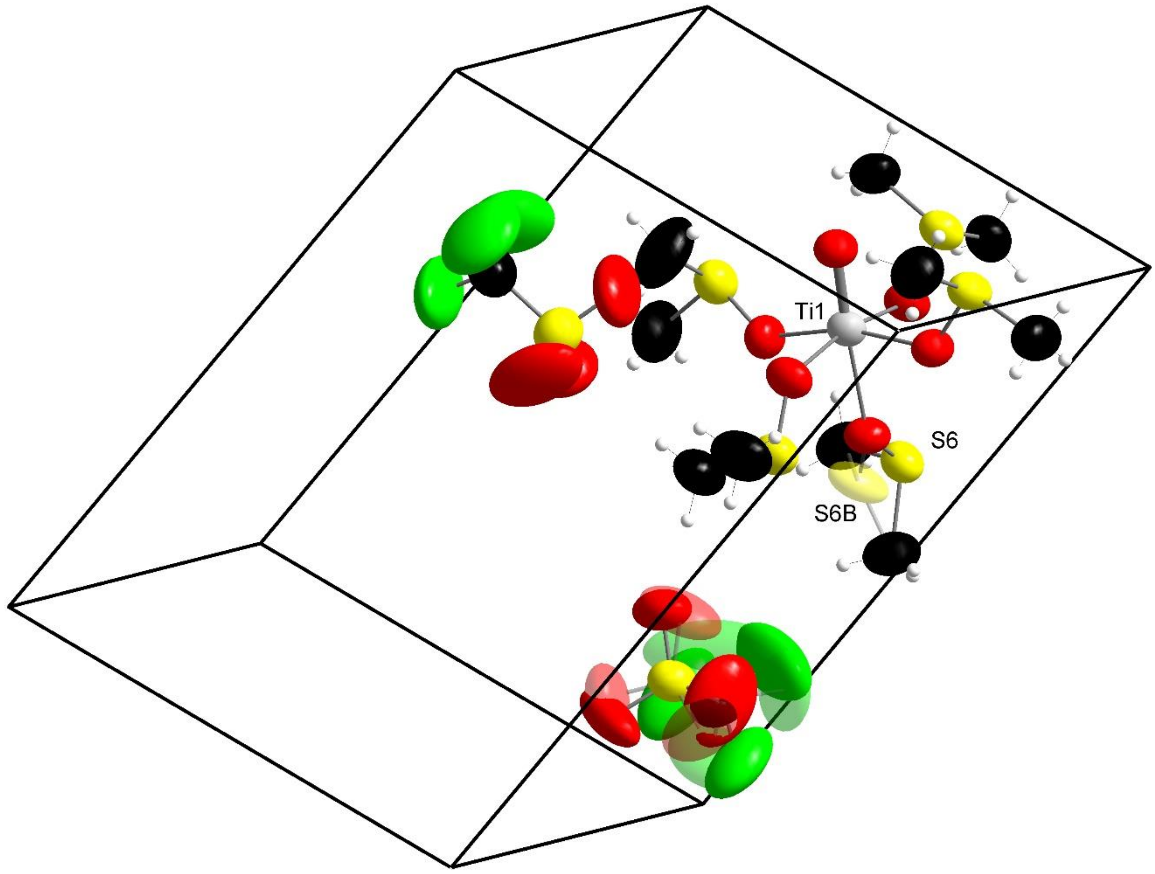

Supplement: Supplementary file 1 — Supplementary material 1 (PDF 260 kb) [file 10953_2017_581_MOESM1_ESM.pdf]
